# Interoceptive NeuroEmpowerment Scale: a trauma-informed measure of adaptive state ownership

**DOI:** 10.3389/fnhum.2026.1748573

**Published:** 2026-04-10

**Authors:** Kate Truitt, McKenna E. Walsh, Michelle R. Dalton

**Affiliations:** 1 Truitt Institute, Pasadena, CA, United States; 2 Department of Family and Community Medicine, School of Medicine, Saint Louis University, Saint Louis, MO, United States

**Keywords:** adaptive state ownership, interoception, neuroplasticity, salience network, self-awareness, trauma neuroscience

## Abstract

The Interoceptive NeuroEmpowerment Scale (INES) is a trauma-informed clinical tool designed to track a person’s moment-to-moment capacity to access and trust adaptive internal states. Rooted in contemporary neuroscience and systems memory theory, the scale offers a real-time, relational alternative to traditional affirmation or distress-based tracking tools. Rather than asking patients to endorse a belief, the INES invites them to rate how much of a chosen resilience state—such as calm, worthiness, or connection—feels internally available in that moment. This percent-based rating is designed to index the momentary accessibility and embodied accessibility of an adaptive internal state, reflecting the extent to which that state is experientially present and tolerable at a given time. Developed in response to stress-associated neuroplastic adaptations and documented negativity bias following trauma exposure, the INES is proposed as a structured method for pacing re-engagement with adaptive internal states within an individual’s regulatory limits. Conceptually grounded in Hebbian plasticity, working memory processes, salience network function, and memory updating frameworks, the scale treats measurement as part of the therapeutic process. This paper outlines the theoretical rationale, proposed mechanisms, standardized administration guidance, and priorities for empirical validation necessary to evaluate the construct of adaptive state ownership.

## 1 Introduction

Interoception refers to the continuous sensing, integration, and interpretation of internal bodily signals that underlie self-awareness and affective experience (Craig, 2009). First described by Sherrington (1906) as the sense of the body’s internal condition, interoception was expanded by Craig (2009) and Craig (2002) to include the cortical representation of visceral signals within the anterior insula, forming the basis for contemporary models of embodied self-awareness. As a construct, interoception encompasses the neural processes that register visceral input from the cardiovascular, respiratory, immune, and gastrointestinal systems and translate these signals into subjectively felt bodily states and emotional salience (Monti et al., 2022; Schmitt and Schoen, 2022). Interoception operates across hierarchically organized brain systems—including the anterior insula, anterior cingulate cortex, and brainstem nuclei—forming a dynamic interface between physiological homeostasis and conscious experience (Chong et al., 2017; Guu et al., 2023). Contemporary network-level work suggests that the embodied sense of self emerges from functional coupling between the salience network and the default mode network (DMN), wherein the anterior insula transforms interoceptive signals into self-referential representations within medial prefrontal and posterior cingulate cortices (Chan et al., 2024; Candia-Rivera et al., 2024). Disturbances within and between these networks contribute to emotional dysregulation and fragmented self-representation across trauma-related and affective disorders, underscoring the clinical relevance of interoceptive awareness as both a diagnostic and therapeutic target (Scalabrini et al., 2022; Schoeller et al., 2024). Within current models of brain–body integration, interoception is conceptualized not simply as detection but as a dynamic, multisystem function supporting homeostatic regulation, self-construction, and adaptive behavioral modulation (Candia-Rivera et al., 2024; Quigley et al., 2021). A schematic of the interoceptive pathway and large-scale network integration relevant to adaptive state ownership is provided in Supplementary Figure S2.

Several instruments have been developed to quantify interoceptive functioning, each emphasizing a distinct dimension of awareness. The Multidimensional Assessment of Interoceptive Awareness (MAIA) (Mehling et al., 2012; Mehling et al., 2018) remains the most widely used self-report tool, capturing noticing, emotional awareness, and self-regulation across eight subscales. Behavioral paradigms such as the Heartbeat Detection Task (Schandry, 1981) and the Heartbeat Discrimination Task (Whitehead et al., 1977) assess objective accuracy in perceiving cardiac signals, whereas the Body Perception Questionnaire (BPQ) (Porges, 1993) and the Body Awareness Questionnaire (BAQ) (Shields et al., 1989) measure subjective sensitivity to autonomic and somatic cues. More recent instruments—the Interoceptive Accuracy Scale (Murphy et al., 2020) and the Cardiac Awareness Questionnaire (Eifert et al., 2000)—target perceived precision and metacognitive confidence, while the Respiratory Interoceptive Attention Task assesses respiratory awareness (Harrison et al., 2021). Collectively, however, these measures chiefly index detection or attention to internal signals rather than the capacity to safely experience and inhabit adaptive internal states.

The Interoceptive NeuroEmpowerment Scale (INES) addresses this theoretical and clinical gap by quantifying the moment-to-moment access to—and ownership of positive affective states and by linking interoceptive awareness to neuroplastic processes that support resilience and self-trust (Supplementary Figure S1). In other words, INES indexes how available an adaptive state feels to the body in the present moment, not whether the state is endorsed as a belief or desired as an ideal. Specifically, it targets a significant, often overlooked challenge in trauma recovery and neuroplastic integration: moving beyond the detection of bodily sensations (interoception) to the ability to experience them as emotionally safe, positive, and inherently one’s own. While traditional measures of interoception (e.g., heartbeat detection tasks, discomfort indexing) emphasize awareness of distress cues (Mehling et al., 2018; Khalsa et al., 2018b), they rarely capture an individual’s capacity to inhabit adaptive internal states—such as calm, confidence, or connection—or to experience those states as trustworthy and self-authored. However, this capacity is what ultimately enables the development of self-awareness, a critical component of conscious experience (Lou et al., 2017; Mograbi et al., 2024). This aligns with findings by Müller-Pinzler et al. (2019), which demonstrate a pronounced negativity bias in self-related belief formation, particularly when individuals perceive opportunities for self-improvement. This pattern is consistent with the broader construct of negativity bias, the tendency to give greater psychological weight to negative over positive experiences, described in detail by Rozin and Royzman (2001). Inwardly focused negativity bias, reinforced by feedback learning, may inhibit the encoding of positive interoceptive states and dampen self-efficacy, highlighting the clinical need for tools like the INES that explicitly track and scaffold ownership of adaptive internal experiences (Furman et al., 2013; Paulus and Stein, 2010; Zhou et al., 2022).

This distinction is critical in trauma-affected nervous systems. Survivors often carry a neurobiological architecture shaped by Stress-Induced Structural Plasticity (SISP) (Bartsch et al., 2023; Vyas et al., 2002; Zhang et al., 2018), which rewires the salience network, amygdala, insula, and hippocampus to over-prioritize threat-based cues and under-integrate positive somatosensory input (Zhang et al., 2018; Bonfanti and Peretto, 2011; Froemke and Young, 2021; Peckham, 2023; Peretto and Bonfanti, 2015; Sahay et al., 2011; Schimmelpfennig et al., 2023; Toda et al., 2019). The interoceptive system (i.e., the insula and anterior cingulate cortex) becomes highly attuned to detecting danger but dulled to signals of safety or well-being (Craig, 2009; Berntson and Khalsa, 2021; Seeley, 2019). As Roeckner et al. (2021) emphasize in their longitudinal imaging review, trauma-exposed individuals show altered connectivity in frontolimbic and salience circuits, impairing affective flexibility and reinforcing maladaptive internal states. As a result, the body becomes an unreliable narrator. Even when external conditions shift, the internal world may still echo fear, shame, or disconnection (Chattarji et al., 2015; McEwen, 2017).

The INES is designed to address this neurobiological bottleneck by tracking embodied access to adaptive emotional states in real time. Measured on a continuum from 0 to 100%, the scale captures not only whether a patient can identify a state like “I feel grounded,” but how much of that state they can feel and own internally—viscerally, cognitively, emotionally, and somatosensorily (Supplementary Figure S2). Crucially, this continuum mirrors the neurocognitive spectrum between state recognition and self-ownership described in the cognitive neuroscience of self-awareness (Lou et al., 2017; Mograbi et al., 2024). Rather than relying on dichotomous self-reporting (e.g., “Do you feel this or not?”), the INES quantifies self-referential integration, providing a psychobiological metric of adaptive state embodiment.

An important nuance of the scale, both clinically and in its underlying neurobiological framework, is the reframing of scores not as achievements but as data: a reflection of the nervous system’s current capacity to safely hold experiences aligned with experiences of safety, belonging, and agency (Truitt, 2025a). These values reflect the biologically embedded mechanisms through which the amygdala assigns emotional and motivational significance (Cunningham and Brosch, 2012; Cunningham and Kirkland, 2014). When the brain detects these values as fulfilled, the autonomic nervous system signals stability (Critchley and Harrison, 2013). When these values are threatened or inaccessible, the brain initiates protective strategies, ranging from hypervigilance to dissociation, to ensure survival. If these values are inconsistently met in early development (due to neglect, trauma, systemic oppression, or betrayal), the brain may begin coding positive internal experiences as foreign or unsafe (Peckham, 2023; de Oliveira Alvares and Do-Monte, 2021).

Clinically, the reframing of scores on the INES as data rather than achievements is a trauma-informed and resilience-focused invitation for patients to honor the moment-to-moment truth of their lived experience and practice separating self from an externally-defined version of reality that many trauma survivors carry. The development and accessibility of interoceptive safety are not solely determined by individual experiences but are also shaped by sociocultural environments. For individuals navigating chronic systemic invalidation (e.g., racism, ableism, gender-based violence, or cultural marginalization), internal cues of safety or worthiness may be implicitly coded as suspect, alien, or unearned. In these contexts, physiological signals once considered markers of well-being may instead evoke internal conflict or hypervigilance, complicating the process of emotional reclamation. The INES accounts for this complexity by offering a non-pathologizing and individually paced structure to rebuild trust in bodily cues—even when those cues have been historically filtered through environments of systemic threat.

For instance:

- Safety may be interpreted as a vulnerability cue, activating freeze instead of regulation.
- Belonging might evoke fear of rejection or invalidation.
- Agency may feel intolerable, producing sabotage or collapse to avoid visibility or comparison.

In alignment with existing literature on valence-weighted feedback learning in which low self-esteem and social anxiety potentiate the weighting of negative over positive self-referential data (Müller-Pinzler et al., 2019), this neurobiological dynamic may exacerbate internal disconfirmation of safety or capability even when conditions are objectively improving.

Recent longitudinal fMRI research demonstrates that interoceptive training enhances connectivity between the anterior insula cortex (AIC) and both bottom-up (nucleus tractus solitarius, brainstem) and top-down (dorsal anterior cingulate cortex, dorsolateral prefrontal cortex, and supramarginal gyrus) structures suggesting that interoceptive ownership is not only sensory but also cognitively co-constructed and regulatory in nature (Berntson and Khalsa, 2021; Sugawara et al., 2024). This is also in alignment with Lou et al. (2017) dual-system model, which situates self-awareness at the intersection of bottom-up salience monitoring and top-down metacognitive integration. The INES is designed to therapeutically bridge these systems, enhancing metacognitive awareness of internal state fluctuations while highlighting the somatosensory basis of cognitive and emotional states.

Rather than reducing interoception to simple bodily detection, the INES builds on the concept described by Chen et al. (2021) of interoception as a full process—one that involves sensing, interpreting, and regulating internal cues to support psychological homeostasis and adaptive functioning. While legacy tools like the Subjective Units of Distress Scale (SUDS) (Wolpe, 1969) remain foundational in tracking real-time distress and are widely validated in exposure-based therapies [for examples see (Kim et al., 2008; Benjamin et al., 2010)], they were not designed to capture the nuances of internal accessibility, trust, or relational proximity to positive or neutral states. The INES serves as a way to track the patient’s readiness to integrate trauma and continue emotional regulation through subjective ownership and enhanced self-awareness. When combined with the SUDS (Wolpe, 1969; Wolpe, 1990), these tools offer a comprehensive view of the patient’s self-efficacy in reducing distress.

Over time, these patterns become embedded in what the brain believes is required for survival. Even joyful or nourishing experiences can be filtered through protective lenses that strip them of their emotional availability. “Feeling good” is no longer simple; it becomes biologically unfamiliar, even dangerous (Apkarian et al., 2011; Lanius et al., 2021; McEwen, 2007). The INES interrupts this loop by providing an opening for reconnection to self without pressure to adapt to externally defined expectations or value structures. It serves as a clinical compass and a vehicle for integration: it helps patients see their nervous system’s readiness in the moment, centers lived experience, and may support engagement of top-down regulation and salience processes through mindful tracking of internal change. INES is designed as a flexible, in-session metric that can be integrated across therapeutic modalities. It indexes the patient’s moment-to-moment adaptive state ownership relative to a patient-selected Resilient Target (Truitt, 2025b)—how internally available and embodied that state feels right now—and functions as collaborative pacing data that supports nervous system–led clinical dialogue, rather than a standalone outcome indicator.

## 2 Theoretical framework

### 2.1 The INES instrument and administration

The INES is a trauma-informed, single-item state rating tool administered on a 0–100% slider. It is designed to track a patient’s moment-to-moment capacity to access and take ownership of a chosen adaptive internal state, rather than to measure distress intensity, symptom severity, or interoceptive detection accuracy (Craig, 2009; Craig, 2002; Quigley et al., 2021). In practice, it functions as both a clinical metric and a structured prompt—an invitation to notice what is biologically available without demanding cognitive compliance.

In the present manuscript, we use the following definitions to clarify scope and terminology. The INES is intended to index adaptive state ownership: the degree to which an adaptive internal state can be accessed and experienced as internally real, embodied, and tolerable in the present moment. The scale is not designed to determine whether a belief is accurate or appropriate, to evaluate performance, or to imply that low scores reflect failure or resistance. Rather, low scores are treated as nervous system data—signals of protective gating, current load, and readiness for integration. This framing is intentional: the INES is meant to support honest tracking of accessibility, not compliance with a desired answer.

We use NeuroEmpowerment to describe a clinically observable shift toward agency and self-trust that can emerge as patients repeatedly contact adaptive internal experience as possible and survivable, even in small increments (Khalsa et al., 2018b; Paulus and Stein, 2010). Inhabiting an internal state refers to felt, bodily access to that state, not merely conceptual understanding. Ownership refers to the patient’s capacity to recognize the state as self-referentially integrated (i.e., “this is present within me and belongs to me”), rather than distant, unsafe, or externally imposed (Craig, 2009; Monti et al., 2022).

Resilient Targets can reflect affective states, relational states, embodied physiological settling, or internally held beliefs that are experienced as lived and tolerable in the body. The INES is designed to track patient-selected adaptive states that are affective, relational, cognitive, and/or somatosensory in nature, including positive and emotionally neutral targets (e.g., “settled,” “secure,” “peaceful,” “strong,” “capable”). Although the slider format could be adapted to rate other internal experiences (e.g., pain, craving, arousal), those uses shift the construct under measurement away from adaptive state ownership and would require independent validation before being interpreted as interoceptive empowerment or resilience accessibility.

#### 2.1.1 Resilient target selection and stability

INES ratings are anchored to a patient-selected Resilient Target: a specific internal experience the patient wants to have more access to (e.g., calm, steadiness, “I’m safe,” connection, capability). In NeuroTriad-based work, Resilient Target development begins only after sufficient stabilization to support interoceptive access without sympathetic override (Paulus and Stein, 2010; Truitt, 2025a). When patients report limited access to adaptive states, this is understood as protective interoceptive gating rather than resistance, particularly in systems in which adaptive affect has historically been paired with vulnerability, loss, or danger (Paulus and Stein, 2010; Truitt, 2025a). To support replication and interpretability, the Resilient Target should be recorded in the patient’s own words at the time it is selected. Once selected, the same Target is ideally held constant for a defined clinical interval to allow change in accessibility to be meaningfully tracked over time.

Targets must be authentic and tolerable. Clinically, this means they are derived from lived moments of genuine access rather than aspirational cognition. Early targets may be emotionally neutral (e.g., “settled,” “steady,” “ordinary”) to reduce threat-based backlash and support successful encoding. As clinical priorities shift or interoceptive capacity increases, Targets may be revised to reflect more specific or nuanced adaptive states. Preliminary thematic clustering of targets in clinical implementation suggests recurring domains of safety, peace/calm, connection/belonging, and empowerment/agency (Walsh et al., 2025).

#### 2.1.2 Anchors and standardized administration language

The INES is anchored from conceptual access to embodied accessibility. The scale may be presented visually (Supplementary Figure S1) or administered verbally. The core intent is the same: the patient is not asked to endorse the state, but to report how available it feels to their system right now. A step-by-step administration protocol is provided in Supplementary Figure S3.

Standardized prompt (verbatim): “Bring to mind your Resilient Target. On a scale from 0 to 100, where 0 means you can understand this state conceptually but you cannot feel it as present in your body right now, and 100 means it feels fully embodied and internally accessible right now, what number fits your experience in this moment?”

Anchor definitions: 0% indicates conceptual knowing without bodily access (“concept only”), whereas 100% indicates full interoceptive accessibility (“fully embodied”). When helpful, clinicians may use brief, non-numeric midpoint descriptors (e.g., “some access,” “partially embodied,” “present but inconsistent”) to support comprehension, while avoiding fixed percentage assignments in order to preserve the scale’s continuous structure.

#### 2.1.3 Clinical workflow and repetition schedule

INES can be used at three time scales. First, it can be sampled within-session: once the patient has returned to regulation, after interventions intended to increase access to the target state, and at session close to support consolidation and collaborative treatment planning. Second, it can be used across sessions to track whether the nervous system is gaining stable, repeated access to the target state over time. Third, when clinically appropriate, brief between-session sampling can support generalization by helping patients notice how context, stress load, relational proximity, and recovery shape state accessibility. In practice, within-session sampling is typically limited to two to four ratings to avoid over-monitoring, while across-session tracking may occur at session start and/or end; between-session sampling may be used briefly (e.g., once daily) during periods of active consolidation or generalization.

The INES can be used flexibly throughout therapy. It is designed to fit within diverse modalities as a state-tracking tool that indexes adaptive state ownership relative to the Resilient Target and, with repeated use, may help strengthen access to that state over time.

#### 2.1.4 Reducing demand characteristics

A key clinically relevant feature of the INES is its non-coercive framing, which permits partial endorsement and ambivalence to be recorded as valid, interpretable response ratings (e.g., 5% or 70%). To reduce “pleasing the clinician” responding, scores are explicitly framed as data, not achievement, and low values are normalized as meaningful clinical information. When useful, the clinician may offer a single brief interoceptive orienting prompt—not as verification, but as anchoring—such as: “*Where do you notice that in your body, if anywhere*?” This approach is consistent with evidence that interoceptive self-report dimensions can dissociate (e.g., attention versus perceived accuracy), underscoring the importance of transparent, non-coercive administration and interpretation (Murphy et al., 2020; Garfinkel et al., 2015). The administration protocol with replicable steps supports consistent use across settings (Supplementary Figure S3).

#### 2.1.5 Response integrity safeguards

Because INES is a self-report rating, it does not provide objective confirmation that a patient is accessing an adaptive internal state at the moment of scoring. Accordingly, the rating is interpreted as the patient’s report of momentary accessibility under the standardized anchors. If the patient indicates they are guessing, selecting a number to satisfy the interviewer, or cannot apply the anchors to their experience, the rating attempt should be paused rather than repeated. The recommended procedure is to (1) restate and clarify the anchors, (2) confirm the Resilient Target as recorded and revise it only if it is not tolerable or not clearly anchored in lived access (documenting any revision as a Resilient Target change), and (3) offer the option to defer the rating (“not rateable right now”) until the patient can respond without pressure.

### 2.2 Mechanism of action and clinical integration

The INES is designed to function as a dynamic, interactive component of the therapeutic process rather than a standalone tool. Its purpose is twofold: (1) to provide a moment-by-moment index of a patient’s capacity to access and take ownership of an adaptive internal state, such as safety or connection—a process grounded in interoceptive awareness and self-agency (Khalsa et al., 2018b; Paulus and Stein, 2010; Farb et al., 2015), and (2) to serve as a scaffold that strengthens these states through repeated, emotionally salient, and relationally anchored experiences that may support neuroplastic updating and repeated access to these states, consistent with models of large-scale network involvement in salience processing and self-referential integration (Lanius et al., 2021; McGaugh, 2015; Seeley et al., 2007).

The scale’s design supports internal self-referencing across multiple neural systems. For instance, the anterior insula (AIC), anterior cingulate cortex (ACC), and dorsolateral prefrontal cortex (dlPFC) combine and mutually mediate interoception, executive function, and emotional regulation (Craig, 2009; Sugawara et al., 2024). In tracking their internal state with the INES, patients may engage the AIC’s integrative process while recruiting the dlPFC to contextualize and organize that state into a coherent narrative. In this way, the act of rating an adaptive state (i.e., “I feel calm,” “I feel worthy”) is regulatory, not just descriptive, aligning with findings that outline the role of metacognitive reflection and bodily self-awareness share in shaping mental state ownership (Lou et al., 2017; Mograbi et al., 2024).

The Default Mode Network (DMN) provides a complementary architecture for understanding why state ownership matters clinically. Once seen as simply a “resting state” system, the DMN is now recognized as a dynamic, integrative network that constructs internal narratives by synthesizing incoming sensory input with stored autobiographical memories, schemas, and beliefs (Yeshurun et al., 2021; Andrews-Hanna et al., 2014; Spreng et al., 2009). Clinically, this is important, as naming an internal state through the INES may interface with the DMN’s ongoing construction of self by holding the state in awareness long enough for it to be integrated into autobiographical meaning-making rather than remaining transiently in working memory (Menon, 2011; Northoff and Bermpohl, 2004). The DMN’s long temporal integration windows assist with binding these experiences into a coherent sense of identity (Northoff and Bermpohl, 2004). Over time, this process may support a gradual shift in self-modeling, such that calm or safety becomes experienced as neurobiologically tangible rather than merely cognitively plausible.

In trauma-affected individuals, this circuitry is often skewed toward hyperactivation of threat pathways and inhibition of top-down modulation (Roeckner et al., 2021). Patients may intellectually know a state like “I am safe” is plausible but experience its internalization as intolerable. This dissonance can be understood as consistent with avoidance learning, which encodes adaptive states as risky due to their previous pairing with vulnerability, exposure, or loss (Peckham, 2023; Wenzel et al., 2018; Remme et al., 2021). Wenzel et al. (2018) demonstrated that phasic dopamine release in the nucleus accumbens, when coupled with endocannabinoid mobilization, can activate avoidance behaviors, even when the original context is no longer threatening. Similarly, trauma-exposed brains may assign aversive salience to otherwise rewarding stimuli, particularly when self-concept is fractured (Pignatelli and Bonci, 2015).

The INES provides a structured space to surface and rewire learned avoidance by normalizing ambivalence and pacing self-trust. Rather than demanding belief in the state, it welcomes partial ownership as valid and trackable (e.g., 5% or 70%), which protects the limbic system from overextension and aligns with trauma-informed and autonomy-supportive care that prioritizes collaboration, pacing, and patient choice over coercion (Elwyn et al., 2012; Deci and Ryan, 2000; Miller and Rollnick, 2013; Substance Abuse and Mental Health Services Administration, 2014). As a patient-reported state measure, the INES emphasizes content clarity, interpretability, and responsiveness; these measurement properties can be evaluated using established patient-reported outcome development standards (Prinsen et al., 2018; Mokkink et al., 2010). Its construct focus is most closely aligned with interoceptive sensibility—defined as self-reported accessibility to and trust in internal states—rather than interoceptive accuracy paradigms (Garfinkel et al., 2015; Khalsa et al., 2018a).

Beneath these higher-order neural networks, the Reticular Activating System (RAS) sets the neurophysiological tone for whether interoceptive access is even available. This brainstem-thalamic system governs overall arousal, filtering incoming sensory data and determining what reaches conscious awareness (Moruzzi and Magoun, 1949; Llinás et al., 1999). When the RAS is dysregulated—such as following chronic threat exposure—patients may struggle to perceive or tolerate positive internal states, even when those states are technically “present.” In such cases, the experiences are not consciously available when the nervous system lacks the arousal capacity or safety signaling to permit integration (Thayer and Lane, 2000; Valentino and Van Bockstaele, 2008). Structural mapping of the ascending reticular activating system (ARAS) reveals a tract extending from the pontine reticular formation to the intralaminar thalamic nuclei, supporting wakefulness and attentional readiness (Yeo et al., 2013; Edlow et al., 2012). The INES, by asking not whether a state is true but whether it’s accessible, honors this biological gatekeeping function—providing a clinical reflection of where the nervous system is currently willing and able to go. This brainstem–thalamic relay and its downstream integration with salience network hubs and the default mode network are summarized in Supplementary Figure S2.

The role of working memory in anchoring these interoceptive shifts is essential, as tracking internal states activates multiple working memory subsystems (Baddeley, 2003), including the phonological loop (when verbalizing the state), visuospatial sketchpad (when visualizing what the state feels like), and episodic buffer (when referencing previous encounters with that state). This scaffolding effect enables transient internal sensations to be held long enough to undergo reframing and reinforcement. When reflecting on changes across sessions (e.g., “Last week, I could only feel 10% calm. Today, it’s 40%”), patients use the working memory system as a cognitive bracket for tracking change. This may be experienced as empowering and may also be biologically strategic insofar as it supports sustained attention to change over time.

By operating across both bottom-up (interoceptive sensation) and top-down (cognitive scaffolding and meaning-making) systems, the INES becomes a flexible adjunct to diverse modalities, including trauma processing, exposure-based treatments, internal family systems work, and somatic-based therapies (Seeley et al., 2007). Its neutral framing allows patients to name internal truths without prematurely affirming them. The focus shifts from belief to capacity: “How available is this state for you in this moment?” This question respects the protective role of the amygdala by honoring its context-sensitive gating of internal threat, and reframes the therapeutic task as one of readiness tracking, not cognitive compliance (Xin et al., 2020; Phelps and LeDoux, 2005). This distinction is essential—traditional cognitive interventions often emphasize restructuring maladaptive thoughts, but belief without embodiment may lead to intellectual assent without felt congruence (Farb et al., 2013). The INES recognizes that a patient may cognitively understand a state is possible (“I know I’m safe now”) while still finding it somatically inaccessible. By emphasizing state accessibility over belief endorsement, the INES bridges the gap between cognition and embodied experience. It helps identify when the nervous system begins to allow what the mind already knows—a shift that marks integration, not performance (Teyler and Rudy, 2007).

The INES is hypothesized to support adaptive salience retuning by repeatedly inviting attention toward internally safe, nourishing, or agency-consistent cues, consistent with models of salience network function in prioritizing interoceptive and exteroceptive information (Menon and Uddin, 2010). Following trauma exposure, this network may misclassify nourishing or safe stimuli as irrelevant—or even threatening—due to maladaptive salience attribution (Phelps and LeDoux, 2005; Liberzon and Martis, 2006; Nicholson et al., 2017). Repeated INES sampling may help make adaptive internal cues more perceptually available and more readily retrievable as lived experiences rather than abstract ideals. Over time and with repetition, these experiences may be more likely to be incorporated into self-referential narratives supported by DMN processes, increasing the felt plausibility of safety, connection, or agency as internal realities rather than aspirational concepts (Craig, 2009; Yeshurun et al., 2021).

### 2.3 The role of measurement in interoceptive ownership and long-term encoding

Whereas traditional interoceptive assessments measure internal awareness of heart rate, breathing, or pain, the INES measures the subjective degree of trust and ownership felt in relation to a chosen adaptive state. In clinical terms, this reflects a patient’s felt-sense partnership with their neurobiology. For example, a 0% rating may mean the state feels conceptually understood but completely unavailable in the body; a 100% rating signals full somatic congruence and emotional embodiment.

This spectrum of accessibility is central to both emotional regulation and memory encoding. Research in memory reconsolidation and systems consolidation highlights that experiences paired with emotional salience, relational cues, and repetition are more likely to be transferred into long-term storage and reactivated in future states of need (McGaugh, 2015; Nader, 2015).

When paired with a Resilient Target—a patient-selected adaptive emotional or somatosensory state intended for cultivation (Truitt, 2025a; Walsh et al., 2025)—or with any other adaptive internal state identified through the therapeutic process, the INES provides a structured way to repeatedly engage internal experience. Each rating can be treated as an index of current accessibility and, over time, may serve as a consistent reference point that supports retrieval of the state in future contexts. Through repeated pairing of adaptive internal experience with stable contextual cues, INES use is hypothesized to support the kinds of rehearsal and salience processes that contribute to longer-term retention and accessibility of adaptive self-states within an individual’s interoceptive and autobiographical landscape (Teyler and Rudy, 2007). From a neuroplasticity perspective, the model is consistent with Hebbian principles often summarized as “neurons that fire together wire together.” When a patient tracks and revisits their internal experience of an adaptive state—particularly across shifting environmental or emotional contexts—repetition may support greater ease of access and familiarity with the associated state representation over time (Remme et al., 2021). Unlike brief cognitive affirmations or binary check-ins, the INES allows for the iterative reinforcement of experience-linked neural connections that support emotional state familiarity, trust, and generalization.

The implications for trauma work could be significant. Patients often cognitively understand how they would like to feel—safe, calm, connected—but the internal experience of those states may remain neurologically out of reach. The INES makes this dissonance visible and trackable without judgment. When a patient indicates 15% ownership of the statement “I am safe,” they are not failing; they are providing a precise, embodied reference point from which reconnection can begin. This simple act may offer the clinician real-time insight into how safety is being evaluated and how much the interoceptive system is currently willing to allow. In this way, the INES becomes more than a measurement tool—it becomes a shared map for attunement, pacing, and ethically grounded collaboration.

This framing is particularly important when comparing the INES to existing subjective measures such as the Subjective Units of Distress Scale (SUDS) (Wolpe, 1969; Wolpe, 1990). While the SUDS has demonstrated construct validity in exposure-based models like EMDR (Kim et al., 2008) and shown consistent predictive patterns in anxiety-focused work (Benjamin et al., 2010), its emphasis remains rooted in distress calibration. It is not designed to track positive interoceptive shifts or the development of ownership of affective safety. The INES is proposed as one way to address this gap by providing an outcome measure for affective accessibility, not just reactivity. In doing so, it expands clinical research capacity into the under-explored space of interoceptive resilience.

Furthermore, the INES can be conceptualized as a fine-grained state-tracking instrument embedded within a dynamic systems framework. Its moment-to-moment measurement structure enables the identification of fluctuations in adaptive state accessibility across time and environmental context. As patients assess their capacity to experience states such as groundedness, worthiness, or connection in varying conditions—clinical settings, social environments, or emotionally charged moments—clinicians can observe where accessibility appears to be strengthening versus where protective avoidance remains prominent. These longitudinal patterns may inform precision treatment planning and the development of individualized practice routines grounded in interoceptive awareness and systems memory principles (Teyler and Rudy, 2007; Kleckner et al., 2017; Ogden and Fisher, 2015).

Finally, interoceptive ownership is not solely an emotional phenomenon—it is also cognitive and somatosensory. Studies by Lou et al. (2017) and Mograbi et al. (2024) indicate that self-awareness is maintained through coordinated activation of medial prefrontal, parietal, and insular networks that link body sensation, narrative processing, and decision-making. The INES may engage these systems by inviting the patient to assign a meaningful, numeric, self-referential value to a felt state. In this way, what is sensed, named, and tracked may become easier to notice, tolerate, trust, and retrieve over time.

## 3 Discussion

### 3.1 A trauma-informed pathway to reclaiming internal truth

For patients with complex trauma, the challenge is rarely in naming a Resilient Target, the emotional or felt-sense ways they would prefer to feel or exist in the world. Most can say, “I want to feel safe” or “I want to believe I belong.” The challenge is rather in allowing that state to land inside the body—to feel it as true, not just imagined. The Interoceptive NeuroEmpowerment Scale provides a structure for tracking and nurturing this process, offering both a mirror for internal experience and a structured way to support forward movement.

Rather than overriding the nervous system, the scale supports attunement, allowing clinicians to monitor when the amygdala is sufficiently regulated for exploration and when additional time or stabilization is required. This neuroethical sensitivity aligns with Roeckner et al.’s (2021) neuroimaging review, which emphasizes the need for flexibility in activating prefrontal and salience networks during trauma recovery. The INES does not seek to prematurely install belief—it invites curiosity about what is currently accessible. In doing so, it disrupts the binary of “believe or not” and instead honors a spectrum: “How much of this truth can you feel right now?”

This is particularly critical when working with trauma-based avoidance. As shown in dopaminergic research, even positive internal states can carry threat value when historically associated with betrayal, loss, or punishment (Wenzel et al., 2018; Pignatelli and Bonci, 2015). These states may be tagged for avoidance through reinforcement-learning dynamics, in which phasic dopamine signaling and endocannabinoid processes contribute to shifting motivational salience away from stimuli that were previously paired with threat or loss. For a survivor, “I feel safe” may light up not reward circuits, but alarm systems. The INES normalizes this, making room for the patient to rate “5% safety” without shame— and, over time, to potentially build tolerance toward increased ownership.

Moreover, the brain’s salience and gating mechanisms are not static. The midline thalamus, as demonstrated by Salay et al. (2018), acts as a flexible relay—assigning behavioral responses based on the interplay between visual, sensory, and interoceptive cues. When a positive state is presented in a therapeutic setting, but the body interprets it through a trauma-conditioned thalamic filter, the nervous system may still select withdrawal or collapse. The INES honors this adaptive protectiveness and offers a regulated space to revisit the signal safely. As Wolff and Vann (2019) note, thalamic circuits are also responsible for updating internal representations, and as such, may be updated over time through slow, safe, repeated exposure to trustworthy interoceptive data that is experienced as trustworthy.

These patterns of protective suppression also tie into how the brain updates its beliefs about self. Müller-Pinzler et al. (2019) describe how negativity bias in belief formation—particularly under social evaluation stress—can lead individuals to down-weight positive self-information in favor of threat-confirming feedback. INES may counter this tendency by enabling a parallel track: instead of asking the patient to cognitively adopt a new belief about themselves, it creates space for a felt experience to be sampled alongside the belief without forcing internal contradiction. It respects the brain’s caution and offers a trauma-informed way to build evidence in partnership with the body.

Within this framework, the INES is proposed as a structured approach to monitoring the momentary accessibility of adaptive internal states, rather than as a mechanism for enforcing cognitive affirmation. Its graded format allows for incremental reporting of accessibility while acknowledging the role of protective regulatory processes in shaping interoceptive access. Lou et al. (2017) and Mograbi et al. (2024) remind us that self-awareness is an emergent property of bodily awareness, emotional context, and reflective integration. The INES draws on all three by helping the patient notice not only what they feel, but what they can feel, when, and how much.

Rather than imposing a therapeutic agenda, the INES becomes a patient-centered, trauma-informed conversation with the nervous system—a question that changes the goal from “fixing” to “partnering.” This process builds biological self-trust, 1 % at a time.

### 3.2 Clinical applications and outcome tracking

In clinical workflow, the INES is most useful when it is embedded at moments of meaningful nervous system transition: after a patient has returned to a regulated baseline, after an intervention designed to increase access to the Resilient Target, and at session close to support consolidation and collaborative planning. Across-session use (e.g., start and/or end of sessions) allows clinicians and patients to track whether adaptive state access is strengthening over time, including durability (how long access persists), context sensitivity (how stress load and relational proximity impact access), and generalization (whether the state becomes retrievable across settings).

In research contexts, INES is well-suited to repeated sampling designs that examine within-person variability in adaptive state accessibility and its relationship to clinical outcomes (Shiffman et al., 2008) For intervention studies, INES can also function as a proximal process marker, testing whether increases in adaptive state ownership mediate downstream change in symptoms, functioning, or engagement, consistent with contemporary frameworks linking interoception and mental health (Quigley et al., 2021; Khalsa et al., 2018a).

Limitations and boundary conditions should be held explicitly. The INES is intentionally minimal (single-item) and therefore should be interpreted as an index of momentary accessibility rather than a comprehensive profile of interoceptive functioning. Ratings may reflect dissociable dimensions of interoception and self-awareness, and interpretability is strengthened when the Resilient Target label and sampling context (e.g., post-regulation versus mid-trigger) are documented alongside the score (Murphy et al., 2020; Garfinkel et al., 2015). Finally, although the slider format can be adapted to track other internal experiences, doing so changes the construct under measurement; those adaptations should not be assumed equivalent to adaptive state ownership without independent validation.

In clinical use, the INES functions as both an assessment tool and a relational connection point between patient and clinician. It supports the collaborative tracking of subtle but significant shifts in a patient’s capacity to experience, trust, and sustain positive affective states—especially those aligned with the amygdala’s core values of safety, belonging, and agency (Seeley, 2019; Truitt, 2025a; Cunningham and Brosch, 2012). The goal is not to reach a fixed score but to build a dynamic awareness of one’s internal truth at any given moment (Lou et al., 2017; Chen et al., 2021).

A score of 0% may reflect a complete absence of somatic awareness or a state that feels cognitively desirable but experientially inaccessible. For patients with complex trauma, neurodevelopmental injury, or identity-based invalidation, such disconnect is not surprising. These patients may report cognitive alignment with a Resilient Target while simultaneously experiencing a somatosensory collapse when attempting to embody it (Peckham, 2023; Roeckner et al., 2021). The INES provides a compassionate metric for this internal gap, allowing patients and clinicians to pace together without pushing the nervous system into overload (Truitt, 2025a).

Conversely, a score approaching 100% suggests full cognitive, somatosensory, and emotional ownership—a moment in which the Resilient Target is not only remembered but inhabited. While rare early in treatment, these scores tend to emerge as therapeutic alliance deepens and adaptive states are repeated in predictable, low-risk environments (Truitt, 2025a; McGaugh, 2015). The INES is designed to make this process trackable.

The trauma-informed utility of the scale lies in its non-coercive structure. It does not require endorsement of internal states that are not currently experientially accessible and permits ambivalent or low ratings to be interpreted as meaningful state data. Consistent with pacing principles in trauma-informed care, ratings are framed as indices of momentary accessibility rather than performance outcomes (Roeckner et al., 2021).

This process is particularly valuable in tracking longitudinal clinical change in adaptive state accessibility. Over the course of weeks or months, clinicians may observe patients increasing in both the frequency and duration of higher interoceptive ownership scores. Early sessions may show fleeting 10–30% resonance with a state like “I feel safe with others.” As practice and repetition accrue, patients may reach 60% within sessions and begin reporting similar states occurring organically in daily life—e.g., in moments of confidence, collaboration, or rest (Sugawara et al., 2024).

Research on subjective distress tracking reveals a comparable predictive arc. For example, Benjamin et al. (2010) demonstrated that distress ratings in anxious youth followed a pattern of intra-session variability and inter-session decrease, suggesting that internal self-report can reliably predict broader regulatory capacity over time. The INES extends this arc by shifting the focus from distress reduction to adaptive state accessibility. This is a critical differentiation. In trauma-impacted patients, tracking distress alone may miss the key variable: the nervous system’s willingness to feel good (Peckham, 2023; Roeckner et al., 2021). Kim et al. (2008) further supported the SUDS’s clinical utility, particularly in exposure-based therapies such as EMDR. However, they also noted that the SUDS lacks granularity in emotional quality, subjective ownership, and directional relevance. The INES offers these features by focusing on how much of an adaptive experience can be embodied—not simply how much of a distressing one can be tolerated.

This inversion positions the INES as a tool for tracking resilience-facing change, rather than solely monitoring distress reduction (Remme et al., 2021). These micro-moments of interoceptive alignment can be clinically meaningful because they mark when a previously fragile internal state becomes more tolerable, more retrievable, and more stable across contexts. When paired with a resilience-oriented therapeutic framework, tools like the INES can help patients track not just progress, but integration—the extent to which a once-fragile internal state becomes a reliable internal resource. In this way, adaptive state tracking becomes more than an assessment: it becomes a structured record of how the nervous system gradually permits greater access to trust, safety, and self-worth (Lou et al., 2017).

### 3.3 Limitations and boundary conditions

Several limitations should be held explicitly when interpreting and applying the INES. First, INES is a single-item, state-based rating tool. This intentional minimalism supports feasibility and repeated sampling, but it also means the scale should be interpreted as an index of momentary accessibility to a specific adaptive state rather than a comprehensive profile of interoceptive functioning (Murphy et al., 2020; Substance Abuse and Mental Health Services Administration, 2014). The INES is therefore not a substitute for multidimensional interoceptive assessment or task-based measures of interoceptive accuracy, nor does it provide direct information about autonomic physiology or neural activity (Craig, 2009; Craig, 2002; Quigley et al., 2021).

Second, interpretability depends on clear target specification and contextual annotation. Because the INES is anchored to a patient-selected Resilient Target, changes in the target label, meaning, or clinical context can shift the construct being rated. For this reason, interpretability is strengthened when the Target is recorded in the patient’s own words and the sampling context is documented alongside the score (e.g., post-regulation, post-intervention, end-of-session; mid-trigger versus baseline) (Murphy et al., 2020; Substance Abuse and Mental Health Services Administration, 2014). When targets are revised, longitudinal comparisons should be treated cautiously unless the revision is explicitly justified as a clinically meaningful refinement rather than a change in the construct under observation.

Third, while the scale is designed to reduce demand characteristics through non-coercive framing, self-report ratings remain vulnerable to social desirability, expectancy effects, and broader common method biases (Orne, 1962; Paulhus, 1991; Podsakoff et al., 2003). The INES should therefore be administered with transparent language emphasizing that low values are informative nervous system data, not failure, and that the goal is tracking accessibility rather than achieving a desired number (Murphy et al., 2020; Substance Abuse and Mental Health Services Administration, 2014). In research contexts, pairing INES ratings with clearly defined sampling schedules and complementary outcome measures may strengthen interpretability and help distinguish state accessibility from general affect or symptom fluctuation (Quigley et al., 2021; Miller and Rollnick, 2013).

Fourth, as noted earlier, although the slider format could be adapted to track other internal experiences (e.g., pain, craving, arousal), such adaptations change the construct under measurement and should not be assumed equivalent to adaptive state ownership without independent validation. The claims advanced in this manuscript are therefore conceptual and hypothesis-generating: the INES is proposed as a tool for tracking adaptive interoceptive accessibility and ownership, with empirical evaluation needed to establish reliability, validity, responsiveness, and meaningful change thresholds across populations and clinical contexts.

### 3.4 Future directions

The INES offers a novel and necessary contribution to trauma-informed care by translating complex neuroscience into an accessible, patient-centred tool for adaptive state tracking. Rather than bypassing the protective strategies in trauma-impacted nervous systems, the scale invites honest dialogue with the body—honouring where safety, belonging, and agency feel available and where they do not. In doing so, it shifts the therapeutic task from installing new beliefs to strengthening what is already biologically possible.

Because the claims of INES are empirically testable, future research should seek to evaluate the tool across both psychometric and mechanistic domains. First, content clarity and response process can be examined through cognitive interviewing with patients and clinicians, ensuring that anchor language (0–100%) is consistently understood and that administration remains non-coercive (FDA, 2009). These interviews can also be used to refine standardized administration scripts and brief protocol steps to maximize replicability across clinicians and research settings. Second, reliability can be evaluated using short-interval test–retest designs appropriate for state-based measures, recognizing that these tools should not be evaluated using trait-based stability assumptions. Third, construct validity can be tested through theoretically coherent associations with established interoception and self-awareness measures (Desmedt et al., 2022), alongside divergence from constructs the INES is not intended to measure (e.g., symptom intensity or belief endorsement) (Murphy et al., 2020; Garfinkel et al., 2015; Khalsa et al., 2018a).

Responsiveness is a central promise of the tool and should be tested explicitly to determine if the INES detects within-session change following interventions designed to increase access to the resilient target and if longitudinal trends correspond with clinically meaningful shifts in functioning and self-trust. Interpretability work can then establish thresholds for clinically meaningful change, distinguishing noise from clinically relevant movement (Prinsen et al., 2018). Finally, mechanistic studies can examine whether INES trajectories correspond with neural and psychophysiological indices implicated in interoception and large-scale network dynamics, consistent with the proposed framework (Craig, 2009; McGaugh, 2015; Seeley et al., 2007; Menon and Uddin, 2010). Together, these steps would allow INES to be evaluated not only as a measure of healing, but as a testable map for how adaptive internal truths become accessible—1 % at a time.

### 3.5 Testable propositions

The model advanced here yields several testable predictions: (1) INES scores will show within-session responsiveness following interventions designed to increase access to the Resilient Target; (2) longitudinal increases in INES will predict functional outcomes and treatment engagement beyond changes in distress alone; (3) INES will demonstrate convergent relationships with interoceptive attention/awareness constructs while remaining dissociable from interoceptive accuracy paradigms; and (4) stable Resilient Targets held across a defined interval will increase interpretability of change trajectories relative to frequently shifting targets. Future research should seek to test these predictions across a variety of settings and populations.

## Data Availability

The original contributions presented in the study are included in the article/Supplementary material, further inquiries can be directed to the corresponding author.

## References

[ref1] Andrews-Hanna J. R. Smallwood J. Spreng R. N. (2014). The default network and self-generated thought: component processes, dynamic control, and clinical relevance. Ann. N. Y. Acad. Sci. 1316, 29–52. doi: 10.1111/nyas.12360, 24502540 PMC4039623

[ref2] Apkarian V. A. Hashmi J. A. Baliki M. N. (2011). Pain and the brain: specificity and plasticity of the brain in clinical chronic pain. Pain 152, S49–S64. doi: 10.1016/j.pain.2010.11.010, 21146929 PMC3045648

[ref3] Baddeley A. (2003). Working memory: looking back and looking forward. Nat. Rev. Neurosci. 4, 829–839. doi: 10.1038/nrn1201, 14523382

[ref4] Bartsch C. J. Jacobs J. T. Mojahed N. Qasem E. Smith M. Caldwell O. . (2023). Visualizing traumatic stress-induced structural plasticity in a medial amygdala pathway using mGRASP. Front. Mol. Neurosci. 16:1313635. doi: 10.3389/fnmol.2023.1313635, 38098941 PMC10720331

[ref5] Benjamin C. L. O'Neil K. A. Crawley S. A. Beidas R. S. Coles M. Kendall P. C. (2010). Patterns and predictors of subjective units of distress in anxious youth. Behav. Cogn. Psychother. 38, 497–504. doi: 10.1017/S1352465810000287, 20509987 PMC4874244

[ref6] Berntson G. G. Khalsa S. S. (2021). Neural circuits of interoception. Trends Neurosci. 44, 17–28. doi: 10.1016/j.tins.2020.09.011, 33378653 PMC8054704

[ref7] Bonfanti L. Peretto P. (2011). Adult neurogenesis in mammals—a theme with many variations. Eur. J. Neurosci. 34, 930–950. doi: 10.1111/j.1460-9568.2011.07832.x, 21929626

[ref8] Candia-Rivera D. Engelen T. Babo-Rebelo M. Salamone P. C. (2024). Interoception, network physiology and the emergence of bodily self-awareness. Neurosci. Biobehav. Rev. 165:105864. doi: 10.1016/j.neubiorev.2024.105864, 39208877

[ref9] Chan A. Harvey P. Hernandez-Cardenache R. Alperin N. Lee S. Hunt C. . (2024). Trauma and the default mode network: review and exploratory study. Front. Behav. Neurosci. 18:1499408. doi: 10.3389/fnbeh.2024.1499408, 39763613 PMC11701030

[ref10] Chattarji S. Tomar A. Suvrathan A. Ghosh S. Rahman M. M. (2015). Neighborhood matters: divergent patterns of stress-induced plasticity across the brain. Nat. Neurosci. 18, 1364–1375. doi: 10.1038/nn.4115, 26404711

[ref11] Chen W. G. Schloesser D. Arensdorf A. M. Simmons J. M. Cui C. Valentino R. . (2021). The emerging science of interoception: sensing, integrating, interpreting, and regulating signals within the self. Trends Neurosci. 44, 3–16. doi: 10.1016/j.tins.2020.10.007, 33378655 PMC7780231

[ref12] Chong J. S. X. Ng G. J. P. Lee S. C. Zhou J. (2017). Salience network connectivity in the insula is associated with individual differences in interoceptive accuracy. Brain Struct. Funct. 222, 1635–1644. doi: 10.1007/s00429-016-1297-7, 27573028

[ref13] Craig A. D. (2002). How do you feel? Interoception: the sense of the physiological condition of the body. Nat. Rev. Neurosci. 3, 655–666. doi: 10.1038/nrn894, 12154366

[ref14] Craig A. D. B. (2009). How do you feel--now? The anterior insula and human awareness. Nat. Rev. Neurosci. 10, 59–70. doi: 10.1038/nrn2555, 19096369

[ref15] Critchley H. D. Harrison N. A. (2013). Visceral influences on brain and behavior. Neuron 77, 624–638. doi: 10.1016/j.neuron.2013.02.008, 23439117

[ref16] Cunningham W. A. Brosch T. (2012). Motivational salience. Curr. Dir. Psychol. Sci. 21, 54–59. doi: 10.1177/0963721411430832

[ref17] Cunningham W. A. Kirkland T. (2014). The joyful, yet balanced, amygdala: moderated responses to positive but not negative stimuli in trait happiness. Soc. Cogn. Affect. Neurosci. 9, 760–766. doi: 10.1093/scan/nst045, 23563851 PMC4040091

[ref18] de Oliveira Alvares L. Do-Monte F. H. (2021). Understanding the dynamic and destiny of memories. Neurosci. Biobehav. Rev. 125, 592–607. doi: 10.1016/j.neubiorev.2021.03.009, 33722616 PMC8783376

[ref19] Deci E. L. Ryan R. M. (2000). The ‘what’ and ‘why’ of goal pursuits: human needs and the self-determination of behavior. Psychol. Inq. 11, 227–268. doi: 10.1207/s15327965pli1104

[ref20] Desmedt O. Heeren A. Corneille O. Luminet O. (2022). What do measures of self-report interoception measure? Insights from a systematic review, latent factor analysis, and network approach. Biol. Psychol. 169:108289. doi: 10.1016/j.biopsycho.2022.108289, 35150768

[ref21] Edlow B. L. Takahashi E. Wu O. Benner T. Dai G. Bu L. . (2012). Neuroanatomic connectivity of the human ascending arousal system critical to consciousness and its disorders. J. Neuropathol. Exp. Neurol. 71, 531–546. doi: 10.1097/NEN.0b013e3182588293, 22592840 PMC3387430

[ref22] Eifert G. H. Thompson R. N. Zvolensky M. J. Edwards K. Frazer N. L. Haddad J. W. . (2000). The cardiac anxiety questionnaire: development and preliminary validity. Behav. Res. Ther. 38, 1039–1053. doi: 10.1016/S0005-7967(99)00132-1, 11004742

[ref23] Elwyn G. Frosch D. Thomson R. Joseph-Williams N. Lloyd A. Kinnersley P. . (2012). Shared decision making: a model for clinical practice. J. Gen. Intern. Med. 27, 1361–1367. doi: 10.1007/s11606-012-2077-6, 22618581 PMC3445676

[ref24] Farb N. Daubenmier J. Price C. J. Gard T. Kerr C. Dunn B. D. . (2015). Interoception, contemplative practice, and health. Front. Psychol. 6:763. doi: 10.3389/fpsyg.2015.00763, 26106345 PMC4460802

[ref25] Farb N. A. S. Segal Z. V. Anderson A. K. (2013). Mindfulness meditation training alters cortical representations of interoceptive attention. Soc. Cogn. Affect. Neurosci. 8, 15–26. doi: 10.1093/scan/nss066, 22689216 PMC3541492

[ref26] FDA (2009). Guidance for Industry Patient-Reported Outcome Measures: Use in Medical Product Development to Support Labeling Claims. Silver Spring, MD: U.S. Department of Health and Human Services, Food and Drug Administration, Center for Drug Evaluation and Research.

[ref27] Froemke R. C. Young L. J. (2021). Oxytocin, neural plasticity, and social behavior. Annu. Rev. Neurosci. 44, 359–381. doi: 10.1146/annurev-neuro-102320-102847, 33823654 PMC8604207

[ref28] Furman D. J. Waugh C. E. Bhattacharjee K. Thompson R. J. Gotlib I. H. (2013). Interoceptive awareness, positive affect, and decision making in major depressive disorder. J. Affect. Disord. 151, 780–785. doi: 10.1016/j.jad.2013.06.044, 23972662 PMC3797260

[ref29] Garfinkel S. N. Seth A. K. Barrett A. B. Suzuki K. Critchley H. D. (2015). Knowing your own heart: distinguishing interoceptive accuracy from interoceptive awareness. Biol. Psychol. 104, 65–74. doi: 10.1016/j.biopsycho.2014.11.004, 25451381

[ref30] Guu S.-F. Chao Y. P. Huang F. Y. Cheng Y. T. Ng H. Y. H. Hsu C. F. . (2023). Interoceptive awareness: MBSR training alters information processing of salience network. Front. Behav. Neurosci. 17:1008086. doi: 10.3389/fnbeh.2023.1008086, 37025109 PMC10070746

[ref31] Harrison O. K. Köchli L. Marino S. Luechinger R. Hennel F. Brand K. . (2021). Interoception of breathing and its relationship with anxiety. Neuron 109, 4080–4093.e8. doi: 10.1016/j.neuron.2021.09.045, 34672986 PMC8691949

[ref32] Khalsa S. S. Adolphs R. Cameron O. G. Critchley H. D. Davenport P. W. Feinstein J. S. . (2018a). Interoception and mental health: a roadmap. Biol. Psychiatry Cogn. Neurosci. Neuroimaging 3, 501–513. doi: 10.1016/j.bpsc.2017.12.004, 29884281 PMC6054486

[ref33] Khalsa S. S. Feinstein J. S. Simmons W. K. Paulus M. P. (2018b). Taking aim at interoception’s role in mental health. Biol. Psychiatry Cogn. Neurosci. Neuroimaging 3, 496–498. doi: 10.1016/j.bpsc.2018.04.007, 29884279 PMC6428192

[ref34] Kim D. Bae H. Chon Park Y. (2008). Validity of the subjective units of disturbance scale in EMDR. J. EMDR Pract. Res. 2, 57–62. doi: 10.1891/1933-3196.2.1.57

[ref35] Kleckner I. R. Zhang J. Touroutoglou A. Chanes L. Xia C. Simmons W. K. . (2017). Evidence for a large-scale brain system supporting allostasis and interoception in humans. Nat. Hum. Behav. 1:0069. doi: 10.1038/s41562-017-0069, 28983518 PMC5624222

[ref36] Lanius R. A. Frewen P. A. Nicholson A. N. McKinnon M. C. (2021). Restoring large scale brain networks in the aftermath of trauma: implications for neuroscientifically-informed treatments. Eur. J. Psychotraumatol. 12:1866410. doi: 10.1080/20008198.2020.1866410 PMC4390556 25854674

[ref37] Liberzon I. Martis B. (2006). Neuroimaging studies of emotional responses in PTSD. Ann. N. Y. Acad. Sci. 1071, 87–109. doi: 10.1196/annals.1364.009, 16891565

[ref38] Llinás R. R. Ribary U. Jeanmonod D. Kronberg E. Mitra P. P. (1999). Thalamocortical dysrhythmia: a neurological and neuropsychiatric syndrome characterized by magnetoencephalography. Proc. Natl. Acad. Sci. USA 96, 15222–15227. doi: 10.1073/pnas.96.26.15222, 10611366 PMC24801

[ref39] Lou H. C. Changeux J. P. Rosenstand A. (2017). Towards a cognitive neuroscience of self-awareness. Neurosci. Biobehav. Rev. 83, 765–773. doi: 10.1016/j.neubiorev.2016.04.004, 27079562

[ref40] McEwen B. S. (2007). Physiology and neurobiology of stress and adaptation: central role of the brain. Physiol. Rev. 87, 873–904. doi: 10.1152/physrev.00041.2006, 17615391

[ref41] McEwen B. S. (2017). Neurobiological and systemic effects of chronic stress. Chronic Stress 1:247054701769232. doi: 10.1177/2470547017692328, 28856337 PMC5573220

[ref42] McGaugh J. L. (2015). Consolidating memories. Annu. Rev. Psychol. 66, 1–24. doi: 10.1146/annurev-psych-010814-014954, 25559113

[ref43] Mehling W. E. Acree M. Stewart A. Silas J. Jones A. (2018). The multidimensional assessment of interoceptive awareness, version 2 (MAIA-2). PLoS One 13:e0208034. doi: 10.1371/journal.pone.0208034, 30513087 PMC6279042

[ref44] Mehling W. E. Price C. Daubenmier J. J. Acree M. Bartmess E. Stewart A. (2012). The multidimensional assessment of interoceptive awareness (MAIA). PLoS One 7:e48230. doi: 10.1371/journal.pone.0048230, 23133619 PMC3486814

[ref45] Menon V. (2011). Large-scale brain networks and psychopathology: a unifying triple network model. Trends Cogn. Sci. 15, 483–506. doi: 10.1016/j.tics.2011.08.003, 21908230

[ref46] Menon V. Uddin L. Q. (2010). Saliency, switching, attention and control: a network model of insula function. Brain Struct. Funct. 214, 655–667. doi: 10.1007/s00429-010-0262-0, 20512370 PMC2899886

[ref47] Miller W. R. Rollnick S. (2013). Motivational Interviewing. New York, NY: Guilford.

[ref48] Mograbi D. C. Hall S. Arantes B. Huntley J. (2024). The cognitive neuroscience of self-awareness: current framework, clinical implications, and future research directions. Wiley Interdiscip. Rev. Cogn. Sci. 15:e1670. doi: 10.1002/wcs.1670, 38043919

[ref49] Mokkink L. B. Terwee C. B. Patrick D. L. Alonso J. Stratford P. W. Knol D. L. . (2010). The COSMIN checklist for assessing the methodological quality of studies on measurement properties of health status measurement instruments: an international Delphi study. Qual. Life Res. 19, 539–549. doi: 10.1007/s11136-010-9606-8, 20169472 PMC2852520

[ref50] Monti A. Porciello G. Panasiti M. S. Aglioti S. M. (2022). The inside of me: interoceptive constraints on the concept of self in neuroscience and clinical psychology. Psychol. Res. 86, 2468–2477. doi: 10.1007/s00426-021-01477-7, 34050431 PMC9674731

[ref51] Moruzzi G. Magoun H. W. (1949). Brain stem reticular formation and activation of the EEG. Electroencephalogr. Clin. Neurophysiol. 1, 455–473. doi: 10.1016/0013-4694(49)90219-9, 18421835

[ref52] Müller-Pinzler L. Czekalla N. Mayer A. V. Stolz D. S. Gazzola V. Keysers C. . (2019). Negativity-bias in forming beliefs about own abilities. Sci. Rep. 9:14416. doi: 10.1038/s41598-019-50821-w, 31594967 PMC6783436

[ref53] Murphy J. Brewer R. Plans D. Khalsa S. S. Catmur C. Bird G. (2020). Testing the independence of self-reported interoceptive accuracy and attention. Q. J. Exp. Psychol. 73, 115–133. doi: 10.1177/1747021819879826, 31519137

[ref54] Nader K. (2015). Reconsolidation and the dynamic nature of memory. Cold Spring Harb. Perspect. Biol. 7:a021782. doi: 10.1101/cshperspect.a021782, 26354895 PMC4588064

[ref55] Nicholson A. A. Rabellino D. Densmore M. Frewen P. A. Paret C. Kluetsch R. . (2017). The neurobiology of emotion regulation in posttraumatic stress disorder: amygdala downregulation via real-time fMRI neurofeedback. Hum. Brain Mapp. 38, 541–560. doi: 10.1002/hbm.23402, 27647695 PMC6866912

[ref56] Northoff G. Bermpohl F. (2004). Cortical midline structures and the self. Trends Cogn. Sci. 8, 102–107. doi: 10.1016/j.tics.2004.01.004, 15301749

[ref57] Ogden P. Fisher J. (2015). Sensorimotor Psychotherapy: Interventions for Trauma and Attachment. New York, NY: W. W. Norton & Company.

[ref58] Orne M. T. (1962). On the social psychology of the psychological experiment: with particular reference to demand characteristics and their implications. Am. Psychol. 17, 776–783. doi: 10.1037/h0043424

[ref59] Paulhus D. L. (1991). “Measurement and control of response Bias,” in Measures of Personality and Social Psychological Attitudes. (San Diego, CA: Academic Press), 17–59.

[ref60] Paulus M. P. Stein M. B. (2010). Interoception in anxiety and depression. Brain Struct. Funct. 214, 451–463. doi: 10.1007/s00429-010-0258-9, 20490545 PMC2886901

[ref61] Peckham H. (2023). Introducing the neuroplastic narrative: a non-pathologizing biological foundation for trauma-informed and adverse childhood experience aware approaches. Front. Psych. 14:1103718. doi: 10.3389/fpsyt.2023.1103718, 37283710 PMC10239852

[ref62] Peretto P. Bonfanti L. (2015). Adult neurogenesis 20 years later: physiological function vs. brain repair. Front. Neurosci. 9:71. doi: 10.3389/fnins.2015.00071, 25798084 PMC4351634

[ref63] Phelps E. A. LeDoux J. E. (2005). Contributions of the amygdala to emotion processing: from animal models to human behavior. Neuron 48, 175–187. doi: 10.1016/j.neuron.2005.09.025, 16242399

[ref64] Pignatelli M. Bonci A. (2015). Role of dopamine neurons in reward and aversion: a synaptic plasticity perspective. Neuron 86, 1145–1157. doi: 10.1016/j.neuron.2015.04.015, 26050034

[ref65] Podsakoff P. M. MacKenzie S. B. Lee J. Y. Podsakoff N. P. (2003). Common method biases in Behavioral research: a critical review of the literature and recommended remedies. J. Appl. Psychol. 88, 879–903. doi: 10.1037/0021-9010.88.5.879, 14516251

[ref66] Porges S. W. (1993). Body Perception Questionnaire. Laboratory of Developmental Assessment. College Park, MD: Laboratory of Developmental Assessment, University of Maryland.

[ref67] Prinsen C. A. Mokkink L. B. Bouter L. M. Alonso J. Patrick D. L. De Vet H. C. . (2018). COSMIN guideline for systematic reviews of patient-reported outcome measures. Qual. Life Res. 27, 1147–1157. doi: 10.1007/s11136-018-1798-3 29435801 PMC5891568

[ref68] Quigley K. S. Kanoski S. Grill W. M. Barrett L. F. Tsakiris M. (2021). Functions of interoception: from energy regulation to experience of the self. Trends Neurosci. 44, 29–38. doi: 10.1016/j.tins.2020.09.008, 33378654 PMC7780233

[ref69] Remme M. W. H. Bergmann U. Alevi D. Schreiber S. Sprekeler H. Kempter R. (2021). Hebbian plasticity in parallel synaptic pathways: a circuit mechanism for systems memory consolidation. PLoS Comput. Biol. 17:e1009681. doi: 10.1371/journal.pcbi.1009681, 34874938 PMC8683039

[ref70] Roeckner A. R. Oliver K. I. Lebois L. A. M. van Rooij S. J. H. Stevens J. S. (2021). Neural contributors to trauma resilience: a review of longitudinal neuroimaging studies. Transl. Psychiatry 11:508. doi: 10.1038/s41398-021-01633-y, 34611129 PMC8492865

[ref71] Rozin P. Royzman E. B. (2001). Negativity bias, negativity dominance, and contagion. Personal. Soc. Psychol. Rev. 5, 296–320. doi: 10.1207/s15327957pspr0504

[ref72] Sahay A. Scobie K. N. Hill A. S. O'Carroll C. M. Kheirbek M. A. Burghardt N. S. . (2011). Increasing adult hippocampal neurogenesis is sufficient to improve pattern separation. Nature 472, 466–470. doi: 10.1038/nature09817, 21460835 PMC3084370

[ref73] Salay L. D. Ishiko N. Huberman A. D. (2018). A midline thalamic circuit determines reactions to visual threat. Nature 557, 183–189. doi: 10.1038/s41586-018-0078-2, 29720647 PMC8442544

[ref74] Scalabrini A. Mucci C. Northoff G. (2022). The nested hierarchy of self and its trauma: in search for a synchronic dynamic and topographical re-organization. Front. Hum. Neurosci. 16:980353. doi: 10.3389/fnhum.2022.980353, 36118976 PMC9478193

[ref75] Schandry R. (1981). Heartbeat perception and emotional experience. Psychophysiology 18, 483–488. doi: 10.1111/j.1469-8986.1981.tb02486.x, 7267933

[ref76] Schimmelpfennig J. Topczewski J. Zajkowski W. Jankowiak-Siuda K. (2023). The role of the salience network in cognitive and affective deficits. Front. Hum. Neurosci. 17:1133367. doi: 10.3389/fnhum.2023.1133367, 37020493 PMC10067884

[ref77] Schmitt C. M. Schoen S. (2022). Interoception: a multi-sensory foundation of participation in daily life. Front. Neurosci. 16:875200. doi: 10.3389/fnins.2022.875200, 35757546 PMC9220286

[ref78] Schoeller F. Horowitz A. H. Jain A. Maes P. Reggente N. Christov-Moore L. . (2024). Interoceptive technologies for psychiatric interventions: from diagnosis to clinical applications. Neurosci. Biobehav. Rev. 156:105478. doi: 10.1016/j.neubiorev.2023.105478, 38007168

[ref79] Seeley W. W. (2019). The salience network: a neural system for perceiving and responding to homeostatic demands. J. Neurosci. 39, 9878–9882. doi: 10.1523/JNEUROSCI.1138-17.2019, 31676604 PMC6978945

[ref80] Seeley W. W. Menon V. Schatzberg A. F. Keller J. Glover G. H. Kenna H. . (2007). Dissociable intrinsic connectivity networks for salience processing and executive control. J. Neurosci. 27, 2349–2356. doi: 10.1523/JNEUROSCI.5587-06.2007, 17329432 PMC2680293

[ref81] Sherrington C. S. (1906). The Integrative Action of the Nervous System. New Haven, CT: Yale University Press.

[ref82] Shields S. A. Mallory M. E. Simon A. (1989). The body awareness questionnaire: reliability and validity. J. Pers. Assess. 53, 802–815. doi: 10.1207/s15327752jpa5304

[ref83] Shiffman S. Stone A. A. Hufford M. R. (2008). Ecological momentary assessment. Annu. Rev. Clin. Psychol. 4, 1–32. doi: 10.1146/annurev.clinpsy.3.022806.091415, 18509902

[ref84] Spreng R. N. Mar R. A. Kim A. S. N. (2009). The common neural basis of autobiographical memory, prospection, navigation, theory of mind, and the default mode: a quantitative meta-analysis. J. Cogn. Neurosci. 21, 489–510. doi: 10.1162/jocn.2008.21029, 18510452

[ref85] Substance Abuse and Mental Health Services Administration. (2014). SAMHSA’S Concept of Trauma and Guidance for a Trauma-Informed Approach. HHS Publication No. (SMA). Rockville, MD: Substance Abuse and Mental Health Services Administration. p. 14–4884.

[ref86] Sugawara A. Katsunuma R. Terasawa Y. Sekiguchi A. (2024). Interoceptive training impacts the neural circuit of the anterior insula cortex. Transl. Psychiatry 14:206. doi: 10.1038/s41398-024-02933-9, 38782961 PMC11116496

[ref87] Teyler T. J. Rudy J. W. (2007). The hippocampal indexing theory and episodic memory: updating the index. Hippocampus 17, 1158–1169. doi: 10.1002/hipo.20350, 17696170

[ref88] Thayer J. F. Lane R. D. (2000). A model of neurovisceral integration in emotion regulation and dysregulation. J. Affect. Disord. 61, 201–216. doi: 10.1016/S0165-0327(00)00338-4, 11163422

[ref89] Toda T. Parylak S. L. Linker S. B. Gage F. H. (2019). The role of adult hippocampal neurogenesis in brain health and disease. Mol. Psychiatry 24, 67–87. doi: 10.1038/s41380-018-0036-2, 29679070 PMC6195869

[ref90] Truitt K. (2025a). The NeuroTriad Model Certification Training. Pasadena, CA: Truitt Institute.

[ref91] Truitt K. (2025b). The NeuroTriad Model: Principles and Practice. Pasadena, CA: Truitt Institute Publishing.

[ref92] Valentino R. J. Van Bockstaele E. (2008). Convergent regulation of locus coeruleus activity as an adaptive response to stress. Eur. J. Pharmacol. 583, 194–203. doi: 10.1016/j.ejphar.2007.11.062, 18255055 PMC2349983

[ref93] Vyas A. Mitra R. Shankaranarayana Rao B. S. Chattarji S. (2002). Chronic stress induces contrasting patterns of dendritic remodeling in hippocampal and amygdaloid neurons. J. Neurosci. 22, 6810–6818. doi: 10.1523/JNEUROSCI.22-15-06810.2002, 12151561 PMC6758130

[ref94] Walsh M. E. Dalton M. Truitt K. Thematic analysis of resilient targets as identified by patients engaging in NeuroTriad model therapy. in The Medical Family Therapy Intensive Conference St. Paul, MN (2025)

[ref95] Wenzel J. M. Oleson E. B. Gove W. N. Cole A. B. Gyawali U. Dantrassy H. M. . (2018). Phasic dopamine signals in the nucleus accumbens that cause active avoidance require endocannabinoid mobilization in the midbrain. Curr. Biol. 28, 1392–1404.e5. doi: 10.1016/j.cub.2018.03.037, 29681476 PMC5940536

[ref96] Whitehead W. E. Drescher V. M. Heiman P. Blackwell B. (1977). Relation of heart rate control to heartbeat perception. Biofeedback Self Regul. 2, 317–392. doi: 10.1007/BF00998623, 612350

[ref97] Wolff M. Vann S. D. (2019). The cognitive thalamus as a gateway to mental representations. J. Neurosci. 39, 3–14. doi: 10.1523/JNEUROSCI.0479-18.2018, 30389839 PMC6325267

[ref98] Wolpe J. (1969). Basic principles and practices of behavior therapy of neuroses. Am. J. Psychiatry 125, 1242–1247. doi: 10.1176/ajp.125.9.1242, 4886238

[ref99] Wolpe J. (1990). The Practice of Behavior Therapy. New York, NY: Pergamon Press.

[ref100] Xin F. Zhou X. Dong D. Zhao Z. Yang X. Wang Q. . (2020). Oxytocin differentially modulates amygdala responses during top-down and bottom-up aversive anticipation. Adv. Sci. 7:2001077. doi: 10.1002/advs.202001077, 32832361 PMC7435249

[ref101] Yeo S. S. Chang P. H. Jang S. H. (2013). The ascending reticular activating system from pontine reticular formation to the thalamus in the human brain. Front. Hum. Neurosci. 7:416. doi: 10.3389/fnhum.2013.00416, 23898258 PMC3722571

[ref102] Yeshurun Y. Nguyen M. Hasson U. (2021). The default mode network: where the idiosyncratic self meets the shared social world. Nat. Rev. Neurosci. 22, 181–192. doi: 10.1038/s41583-020-00420-w, 33483717 PMC7959111

[ref103] Zhang X. Ge T. T. Yin G. Cui R. Zhao G. Yang W. (2018). Stress-induced functional alterations in amygdala: implications for neuropsychiatric diseases. Front. Neurosci. 12:367. doi: 10.3389/fnins.2018.00367, 29896088 PMC5987037

[ref104] Zhou H. Zou H. Dai Z. Zhao S. Hua L. Xia Y. . (2022). Interoception dysfunction contributes to the negative emotional bias in major depressive disorder. Front. Psych. 13:874859. doi: 10.3389/fpsyt.2022.874859, 35479498 PMC9035634

