# Supplementary material for: Interoceptive NeuroEmpowerment Scale: a trauma-informed measure of adaptive state ownership
Source: Front Hum Neurosci. 2026 Apr 10;20:1748573. doi: 10.3389/fnhum.2026.1748573 (PMC13106559; doi:10.3389/fnhum.2026.1748573)
Supplement: Supplementary file 1 [file Data_Sheet_1.pdf]

Supplementary Material

1 Supplementary Figures

Supplementary Figure S1. INES Rating Slider — Resilient Target (example: Calm)

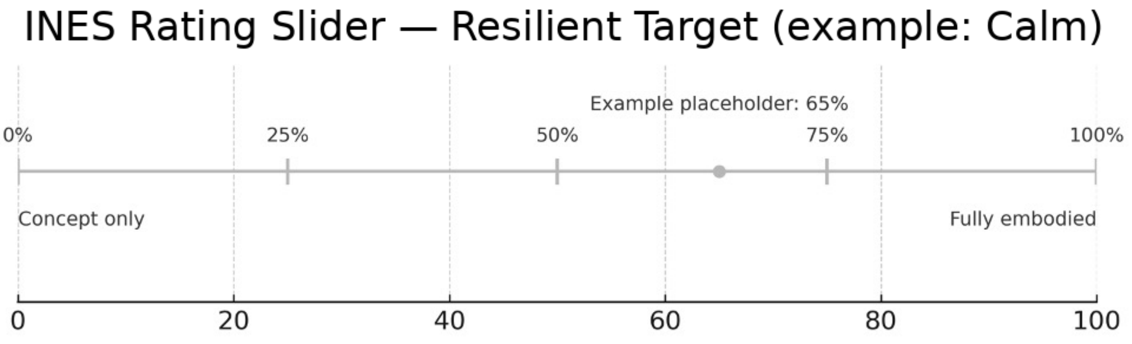

The Interoceptive NeuroEmpowerment Scale (INES) slider represents a continuous 0–100% rating of momentary accessibility to a specified adaptive internal state (the "Resilient Target"). A rating of 0% denotes conceptual understanding without bodily access ("concept only"), whereas 100% denotes full interoceptive accessibility ("fully embodied"). Tick marks, when shown, are provided for visual reference only and do not indicate discrete levels or cut scores. Ratings may be sampled within and across sessions to examine patterns of adaptive state accessibility over time.

Supplementary Figure S2. INES Conceptual Model

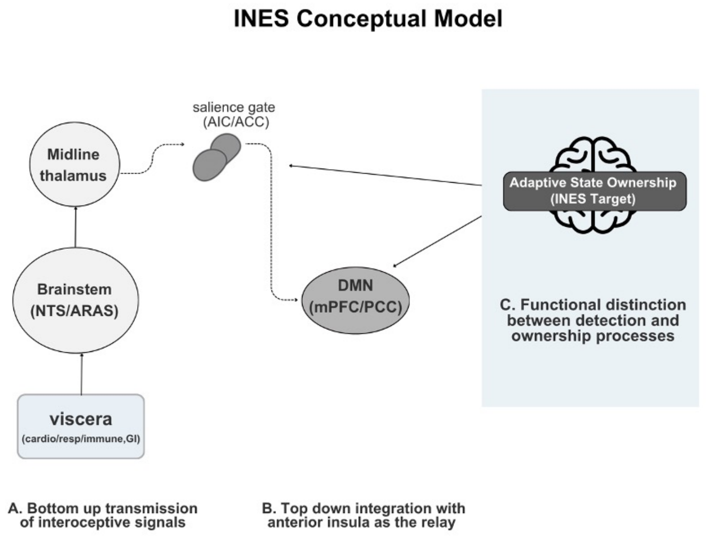

Left-to-right schematic linking bottom-up interoceptive inputs from viscera through brainstem nuclei (including NTS/ARAS) to the midline thalamus, with subsequent relay to salience network hubs (AIC/ACC) and integration with the default mode network (mPFC/PCC). This model illustrates where interoceptive input becomes an adaptive, self-referential internal state—i.e., adaptive state ownership—functionally distinct from earlier sensory detection processes.

Supplementary Figure S3. INES Administration Protocol

Administration protocol (replicable steps):
To support consistent use across clinicians and research settings, INES administration may follow a brief, standardized sequence:

1) Confirm sufficient stabilization (i.e., the patient can orient to internal experience without sympathetic override).
2) Identify and document the Resilient Target in the patient's own words (e.g., "calm," "present," "safe," "connected," "capable").
3) Deliver the standardized INES prompt and obtain a 0–100% rating.
4) Record the score *alongside the exact Target label and the sampling context/timepoint* (e.g., post-regulation, post-intervention, end-of-session) to strengthen interpretability.
5) If clinically useful, offer one non-verifying interoceptive orienting question (e.g., *"Where do you notice that experience of [Resilient Target], if anywhere?")* to support anchoring rather than performance.

Use the score as collaborative pacing data.

Sample clinical language for Resilient Target identification:
"Before we use the INES, let's choose a Resilient Target—a word or short phrase for an internal state you'd like more access to. It should feel possible, even in a small amount (even 1%). If nothing feels accessible yet, we can start with something neutral like 'settled' or 'present.' What word or phrase fits best for you right now? We'll write that down and use it for the rating."

INES administration protocol steps are provided to promote consistent use across settings.
